# Genome-Wide Expression Profiling of mRNAs, lncRNAs and circRNAs in Skeletal Muscle of Two Different Pig Breeds

**DOI:** 10.3390/ani11113169

**Published:** 2021-11-05

**Authors:** Xinhua Hou, Ligang Wang, Fuping Zhao, Xin Liu, Hongmei Gao, Lijun Shi, Hua Yan, Lixian Wang, Longchao Zhang

**Affiliations:** Institute of Animal Science, Chinese Academy of Agricultural Sciences, Beijing 100193, China; 7hxh73@163.com (X.H.); ligwang@126.com (L.W.); zhaofuping@caas.cn (F.Z.); firstliuxin@163.com (X.L.); gaohongmei_123@126.com (H.G.); shilijun01@caas.cn (L.S.); zcyyh@126.com (H.Y.); iaswlx@263.net (L.W.)

**Keywords:** pigs, RNA-seq, skeletal muscle, mRNAs, lncRNAs, circRNAs

## Abstract

**Simple Summary:**

Variation exists in muscle-related traits, such as muscle growth and meat quality, between obese and lean pigs. In this study, the transcriptome profiles of skeletal muscle between Beijing Blackand Yorkshire pigs were characterized to explore the molecular mechanism underlying skeletal muscle-relatedtraits. Gene Ontology (GO) and KEGG pathway enrichment analyses showed that differentially expressed mRNAs, lncRNAs, and circRNAs involved in skeletal muscle development and fatty acid metabolism played a key role in the determination of muscle-related traits between different pig breeds. These results provide candidate genes responsible for muscle phenotypic variation and are valuable for pig breeding.

**Abstract:**

RNA-Seq technology is widely used to analyze global changes in the transcriptome and investigate the influence on relevant phenotypic traits. Beijing Black pigs show differences in growth rate and meat quality compared to western pig breeds. However, the molecular mechanisms responsible for such phenotypic differences remain unknown. In this study, *longissimus dorsi* muscles from Beijing Black and Yorkshire pigs were used to construct RNA libraries and perform RNA-seq. Significantly different expressions were observed in 1051 mRNAs, 322 lncRNAs, and 82 circRNAs. GO and KEGG pathway annotation showed that differentially expressed mRNAs participated in skeletal muscle development and fatty acid metabolism, which determined the muscle-related traits. To explore the regulatory role of lncRNAs, the *cis* and *trans*-target genes were predicted and these lncRNAswere involved in the biological processes related to skeletal muscle development and fatty acid metabolismvia their target genes. CircRNAs play a ceRNA role by binding to miRNAs. Therefore, the potential miRNAs of differentially expressed circRNAs were predicted and interaction networks among circRNAs, miRNAs, and key regulatory mRNAs were constructed to illustrate the function of circRNAs underlying skeletal muscle development and fatty acid metabolism. This study provides new clues for elucidating muscle phenotypic variation in pigs.

## 1. Introduction

Pigs are a major source of meat production for human consumption. To increase meat yields and reduce costs, lean pig breeds are under intensive selection and exhibit a fast growth rate, improved feed efficiency, and more lean meat content compared to obese pig breeds. However, long-term selection leads to deterioration in the meat quality of lean pig breeds. Numerous studies suggest that skeletal muscle characteristics, such as myofiber diameter, myofiber density, and intramuscular fat content (IMF), are different between lean and obese pig breeds [1,2,3,4,5,6]. Muscle mass, plus size, number, area, and density of muscle fibers not only reflect skeletal muscle development but also determine meat quality [7]. IMF content is associated with meat tenderness, flavor, and juiciness and is crucial for meat quality [8]. Meat quality can be improved by increasing the IMF content based on intramuscular adipogenesis [2].

As the genetic background is a key factor affecting animal phenotype, exploration of the molecular mechanisms mediating skeletal muscle development and lipid deposition are vital to genetic breeding for lean meat percentage and meat quality [9]. High-throughput RNA-seq is a powerful approach to estimate the transcriptome profile, detect a transcript expressed at a low level, identify post-transcriptional mutations, and analyze alternative splice sites or isoforms. The RNA-seq method has already been used to explore the transcriptome profile, including coding genes, long non-coding RNAs (lncRNAs), miRNAs, and circular RNAs (circRNAs), among the different pig breeds [3,4,10,11].

The Beijing Black pig was developed in China and is characterized by high IMF content, slow-growing muscle, low body weight, and superior meat quality compared to western lean pig breeds. However, it is unclear about the regulatory mechanism of those breed-specific differences. In this study, the transcriptome profiles of skeletal muscle tissues were analyzed via the RNA-seq method to further decipher the molecular mechanism underlying skeletal muscle growth and meat quality between Beijing Black pigs and Yorkshire pigs. This study identified key candidate genes and provided useful information for porcine genetic improvement for lean meat percentage and meat quality.

## 2. Materials and Methods

### 2.1. Animals and Tissue Samples

Four Beijing Black pigs and four Yorkshire pigs were rendered unconscious by electrical stunning and slaughtered in the local abattoir. All of the pigs were barrows and were harvested at 210 days of age. The samples of *longissimus* dorsi muscles were collected and frozen immediately in liquid nitrogen for RNA sequencing.

### 2.2. RNA Isolation and Library Construction

Total RNA was prepared by TRIzol reagent (Invitrogen, CA, USA) following the manual instructions. Electrophoresis on 1% agarose gels was performed to monitor the RNA degradation and contamination. The concentration and purity of RNA were measured by Nanodrop. The RNA integrity was detected by Agilent 2100 bioanalyzer. An amount of 3 μg RNA was used for removal of ribosomal RNA (rRNA) by EpicentreRibo-zero™ rRNA Removal Kit (Epicentre, Madison, WI, USA). The rRNA-depleted RNA was randomly broken into the fragments within 250–300 bp. Random hexamer primer and M-MuLV Reverse Transcriptase were used to synthesize the first-strand cDNA, and second-strand cDNA was obtained by DNA Polymerase subsequently. Then, the fragments were ligated to NEBNext Adaptor and purified with AMPure XP system (Beckman Coulter, Beverly, MA, USA). Finally, the library fragments were augmented by PCR with Phusion High-Fidelity DNA polymerase.

### 2.3. Illumina Sequencing and Quantification

Eight cDNA libraries were sequenced on an Illumina HiSeq 2500 platform. After removing adapter reads, poly-N containing reads, and low-quality reads, clean data were obtained for Q20, Q30, and GC content assessment and following analysis. Hisat2 (v2.1.0) was used to build an index of the porcine reference genome (Sscrofa 11.1) by its build-index function and to align the clean reads to the reference genome with default parameters. The Pearson correlation analysis of all the samples was performed by read counts from the top 1000 differentially expressed transcripts. The mapped reads were assembled by Stringtie (v1.3.5) and reconstructed transcripts were merged by Cuffmercge (Cufflinks v2.2.1). Fragments per kb for a million reads (FPKM) of each gene were calculated according to the length and read counts of this gene. The transcripts located on the Y chromosome were removed to eliminate the sex effect. Differentially expressed genes between Beijing Black and Yorkshire pigs were parsed by edgeR package. Genes with q value < 0.05 and fold change (FC) ≥ 2 were selected for significant differential expression.

### 2.4. Identification of Novel lncRNAs and Targets Prediction

The nonannotated transcripts were used to identify novel lncRNAs. After discarding the transcripts with length less than 200 nt, the protein-coding potential was estimated using Coding Potential Calculator (v0.9-r2), Pfam-scan (v1.3), and Coding-Non-CodingIndex (v2). The transcripts which had no coding potential were considered as candidate novel lncRNAs. LncRNAs can regulate their target genes in a *cis* (co-location) or *trans* (co-expression) manner. The neighboring genes from 100 kb upstream or downstream of a lncRNA were considered as its *cis*-target genes. *Trans*-target genes were predicted by the Pearson correlation between the expression levels of lncRNAs and mRNAs with the criteria of *p* value < 0.05 and |R| > 0.9.

### 2.5. CircRNA Prediction and Biological Analysis

All the unmapped reads were used to identify circRNAs by both CIRI (v 2.0.6) and find_circ (v 1.0) [12,13]. The miRNA binding site prediction was conducted using the miRanda algorithms. Transcripts per million reads (TPM) were used to normalize the raw counts and differential expression analysis was performed by DESeq R package with q value < 0.05 and fold change ≥ 2.

### 2.6. Gene Ontology (GO) Term and KEGG Pathway Enrichment Analysis

GO and KEGG pathway enrichment analysis was implemented by the DAVID Gene Ontology database (accessed on 2 June 2021; https://david.ncifcrf.gov/). Each term with a *p* value < 0.05 was considered significantly enriched.

## 3. Results

### 3.1. Overview of Sequencing Data

A comparative transcriptomic analysis was conducted to better understand the molecular mechanism for the different phenotypes of skeletal muscles between Beijing Black and Yorkshire pigs. More than 88 million clean reads were obtained from each library. The GC contents ranged from 51.87% to 56.15%. Each sample had a Q20 > 93.9% and a Q30 > 85.8%. More than 90% of the clean reads were mapped to the reference genome and 77.01% to 82.59% of those were uniquely mapped (Table S1 and Table S2). About 59.9–72.9% of the reads mapped to exonic regions, 11.95–18.06% to intronic regions, and 15.14–22.02% to intergenic regions (Figure S1). The samples showed a high correlation within the Beijing Black and Yorkshire breeds (ranged from 0.64 to 0.73), while the correlation between the two pig breeds was poor (ranged from 0.46 to 0.56) (Figure S2). These results suggested that four samples in each breed showed appropriate consistency and representativeness.

### 3.2. Identification of lncRNAs in Porcine Skeletal Muscle

The transcripts with no annotation information were used for novel lncRNA analysis. The protein-coding potential was evaluated by CPC, CNCI, and PFAM. A set of 7911 transcripts remained at the intersection of the three methods (Figure S3A). Finally, the 3448 lncRNAs were obtained by removing single-exon or short transcripts and also those with coding potential (Table S3). The average length of lncRNAs was shorter than that of mRNAs (Figure S3B), and lncRNAs tended to contain fewer exons than mRNAs (Figure S3C). The lncRNAs also tended to be shorter in ORF length than mRNAs (Figure S3D). Those results were similar to the previous reports [14,15].

### 3.3. CircRNA Detection in Porcine Skeletal Muscle

CircRNAs were predicted by CIRI and find_circ. As a result, a total of 18,217 candidate circRNAs were identified, 2631 of which existed in all the samples (Table S4). The length of circRNAs ranged from 22 to 1436 nt, and the average length was 286 nt (Figure S4A). The majority of circ RNAs (88.7–90.3%) were exoniccircRNAs, 4.8–5.6% were intronic circRNAs, and 4.7–5.7% were intergenic circRNAs (Figure S4B). Genomic mapping revealed that these circRNAs were distributed widely on the pig chromosomes with the highest number (2098; 11.5%) on chromosome 1 (Figure S4C).

### 3.4. Differential Expression Analysis

**Figure 1:**
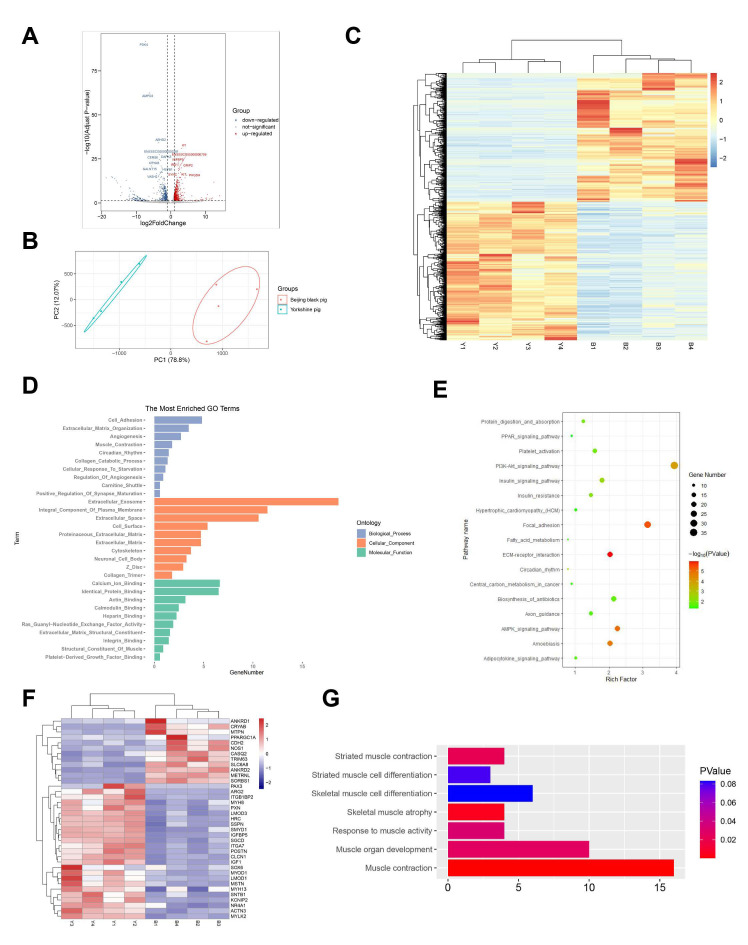
Expression profiles and functional analysis of differentially expressed mRNAs. (**A**) Volcano plot of differentially expressed mRNAs between Beijing Black and Yorkshire pigs. The up- and downregulated mRNAs are shown in red and blue, separately. (**B**) Principal component analysis of eight skeletal muscle samples by differentially expressed mRNAs. (**C**) Heat map of differentially expressed mRNAs in skeletal muscle. Yellow shows higher expression and blue shows lower expression. (**D**) GO enrichment of differentially expressed mRNAs. (**E**) KEGG pathway annotation of differentially expressed mRNAs. (**F**) Heat map of 37 differentially expressed genes related to skeletal muscle-related traits. (**G**) GO terms classification of 37 differentially expressed genes related to skeletal muscle-related traits.

**Figure 2:**
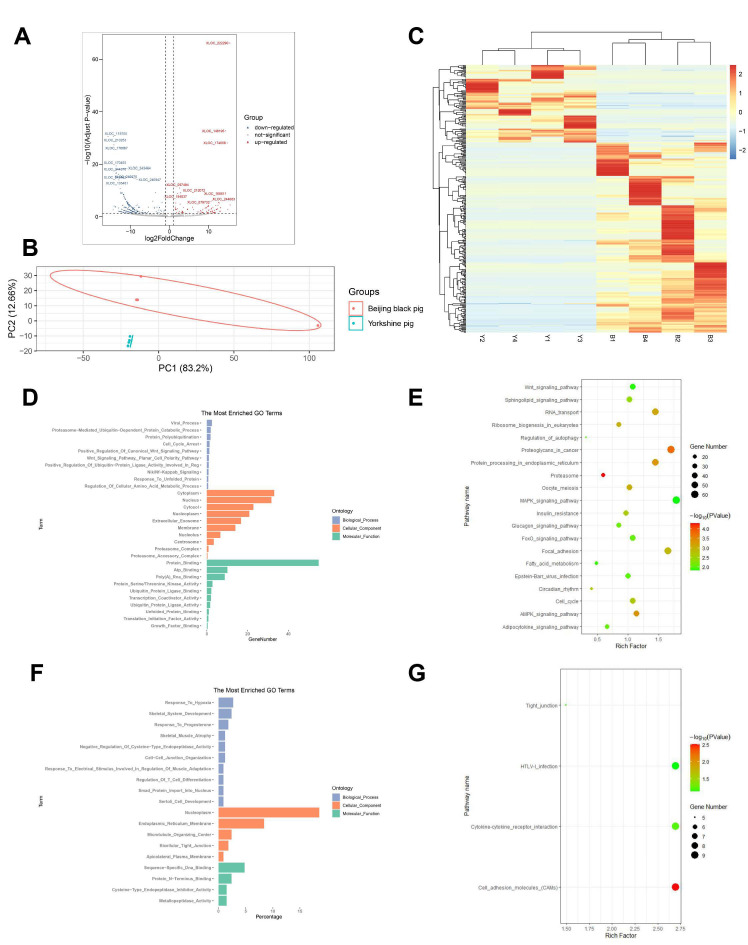
Expression profiles and functional analysis of differentially expressed lncRNAs. (**A**) Volcano plot of differentially expressed lncRNAs between Beijing Black and Yorkshire pigs. The up- and downregulated lncRNAs are shown in red and blue, separately. (**B**) Principal component analysis of eight skeletal muscle samples by differentially expressed lncRNAs. (**C**) Heat map of differentially expressed lncRNAs in skeletal muscle. Yellow shows higher expression and blue shows lower expression. (**D**) GO enrichment of the *cis*-target genes regulated by differentially expressed lncRNAs. (**E**) GO enrichment of the *trans*-target genes regulated by differentially expressed lncRNAs. (**F**) KEGG pathway annotation of the *cis*-target genes regulated by differentially expressed lncRNAs. (**G**) KEGG pathway annotation of the *trans*-target genes regulated by differentially expressed lncRNAs.

**Figure 3:**
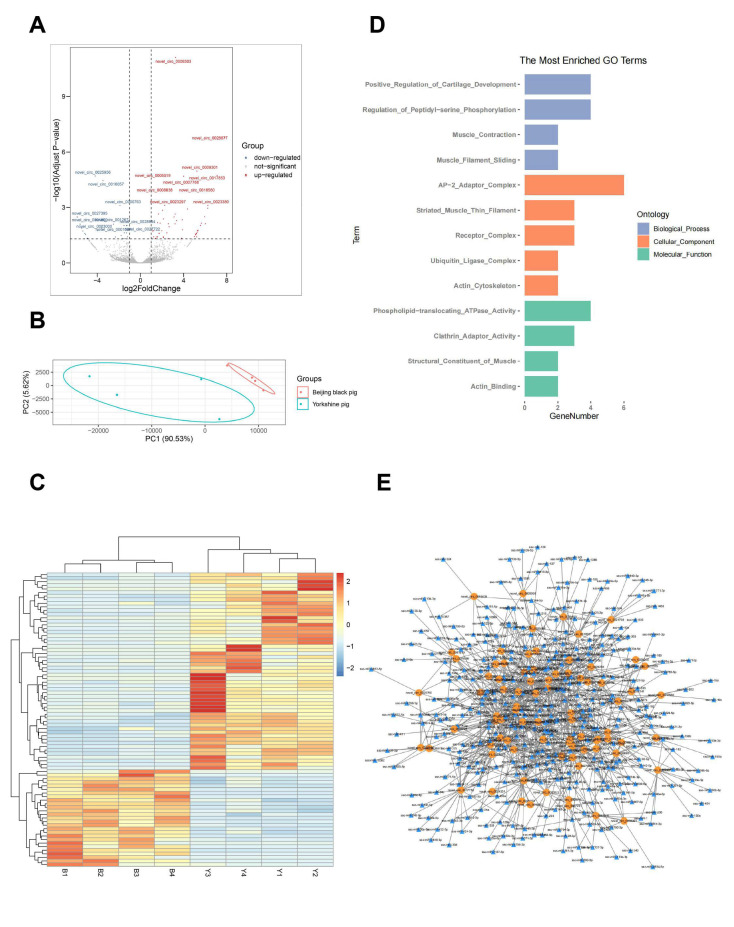
Expression profiles and functional analysis of differentially expressed circRNAs. (**A**) Volcano plot of differentially expressed circRNAs between Beijing Black and Yorkshire pigs. The up- and downregulated circRNAs are shown in red and blue, separately. (**B**) Principal component analysis of eight skeletal muscle samples by differentially expressed circRNAs. (**C**) Heat map of differentially expressed circRNAs in skeletal muscle. Yellow shows higher expression and blue shows lower expression. (**D**) GO enrichment of the host genes generating differentially expressed circRNAs. (**E**) The interaction network between differentially expressed circRNAs and their predicted miRNAs.

For mRNAs and lncRNAs, expression levels were normalized as FPKM, while TPM was used to quantify the expression levels of circRNAs. The criteria used for the identification of differentially expressed genes were log2|FC| > 1 and q value < 0.05. Finally, 1051 mRNAs (550 upregulated and 501 downregulated), 322 lncRNAs (95 upregulated and 227 downregulated), and 82 circRNAs (55 upregulated and 27 downregulated) were identified in the comparison of Beijing Black vs. Yorkshire pigs (Table S5, Figure 1A, Figure 2A and Figure 3A). Principal component analysis elucidated that PC1 accounted for 78.8%, 83.2%, and 90.53% of differentially expressed mRNAs, lncRNAs, and circRNAs, while PC2 accounted for 12.07%, 12.66%, and 5.62%, respectively (Figure 1B, Figure 2B and Figure 3B). The heatmap plots revealed that differentially expressed genes could be used to classify the samples into two groups that were consistent with each breed (Figure 1C, Figure 2C and Figure 3C).

### 3.5. Functional Analysis of Transcriptome Data

GO and KEGG enrichment analysis were performed to shed light on the potential function of differentially expressed mRNAs concerned with skeletal muscle-related traits between Beijing Black and Yorkshire pigs. The top significantly enriched GO terms of the biological process included extracellular matrix organization, cell adhesion, and muscle contraction. (Figure 1D, Table S6). KEGG enrichment analysis revealed that the differentially expressed mRNAs were mainly enriched in the ECM-receptor interaction, focal adhesion, AMPK signaling pathway, PI3K-Akt signaling pathway, Adipocytokine signaling pathway, fatty acid metabolism, and PPAR signaling pathway (Figure 1E, Table S6). Some differentially expressed genes, such as TRIM63, CRYAB, MSTN, MYLK2, MYOD1, PAX3, IGF1, SOX6, and ITGB1BP2, were engaged in the GO terms related to muscle contraction, skeletal muscle atrophy, striated muscle contraction, muscle organ development, and response to muscle activity (Figure 1F,G). These genes reflected that Yorkshire and Beijing Black pigs had different characteristics in development and physiology. As those two pig breeds present different IMF content, the GO terms referring to fatty acid metabolism or adipogenesis were the main points of focus. Differentially expressed genes, such as FABP3, PDK4, LDLR, APOE, APOB, CPT1A, and CPT1B, were involved in fatty acid transport, oxidation, and biosynthesis and considered as key candidate genes in the regulation of IMF content.

The potential *cis* and *trans*-target genes were predicted to investigate the function of differentially expressed lncRNAs (Table S7). As a result, 483 *cis*-target and 4173 *trans*-target genes were obtained. GO and KEGG enrichment analysis revealed that *cis*-target genes are involved in the biological processes including skeletal muscle atrophy, cell-cell junction organization, and myoblast differentiation, while *trans*-target genes are involved in cell cycle arrest, MAPK cascade, skeletal muscle tissue development, fatty acid beta-oxidation and biosynthesis, and adipose tissue development (Figure 2D,F, Table S8), which were related to the skeletal muscle development and IMF formation. The *trans*-target genes took part in the AMPK signaling pathway, FoxO signaling pathway, MAPK signaling pathway, and PI3K-Akt signaling pathway, while *cis*-target genes only significantly took part in cell adhesion molecules according to the result of KEGG enrichment (Figure 2E,G, Table S8).

**Figure 4:**
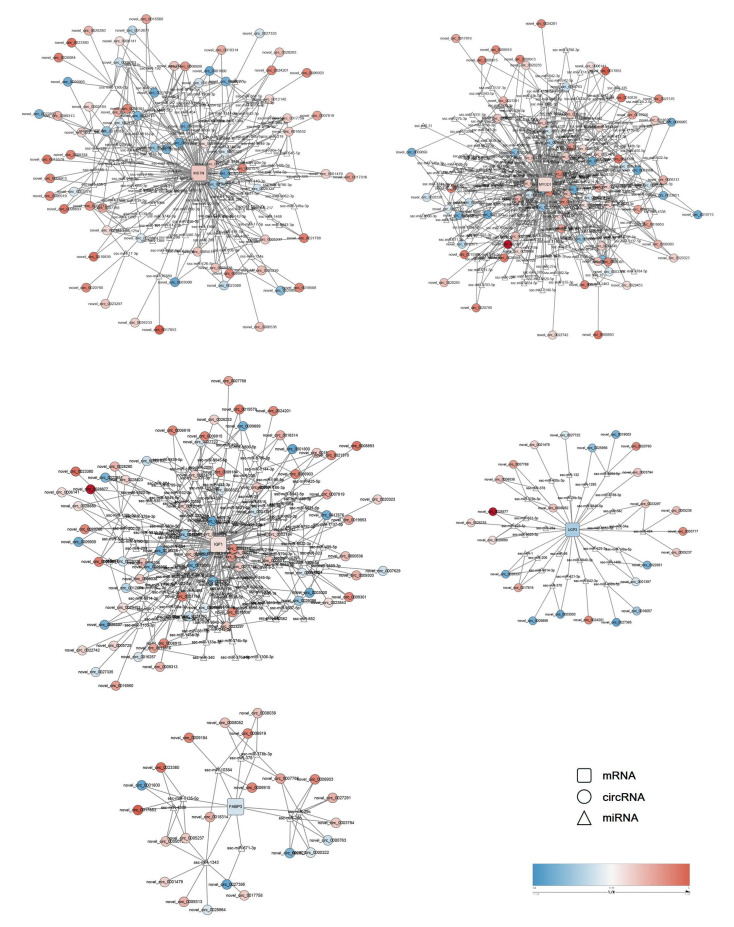
CeRNA network of key candidate regulatory genes for skeletal muscle development and fatty acid metabolism. Rounded rectangles indicate candidate regulatory genes. Circles indicate circRNAs. Triangles indicate miRNAs. Blue indicates downregulated expression and red indicates upregulated expression.

To elucidate the potential function of circRNAs in the skeletal muscles between the different pig breeds, the GO and KEGG enrichment analysis of the host genes which produced differentially expressed circRNAs was carried out. The results showed that these host genes were significantly assigned to the biological processes of muscle contraction and muscle filament sliding (Figure 3D, Table S9). CircRNA is a kind of competing endogenous RNA (ceRNA), which can sponge the miRNAs and remove the inhibition of miRNA on their target genes [16]. Therefore, the prediction of the interaction between the differentially expressed circRNAs and miRNAs was performed to illustrate the ceRNA roles of these circRNAs. Finally, 1008 circRNA-miRNA interaction pairs were predicted for 82 differentially expressed circRNAs and 342 miRNAs (Figure 3E, Table S10). Additionally, differentially expressed MSTN, IGF1, MYOD1, FABP3, and UCP3 between the two pig breeds were the key regulators for skeletal muscle development and fatty acid metabolism [17,18,19,20,21]. Therefore, the circRNA–miRNA–mRNA regulatory networks were outlined to define the potential ceRNA roles of circRNAs on these key genes in the regulation of skeletalmuscle-related traits between the two pig breeds (Figure 4).

As the biological processes concerning muscle characteristics and energy metabolism were vital in the determination of the skeletal muscle phenotype between Beijing Black and Yorkshire pigs, the interaction networks of differentially expressed mRNAs, lncRNAs, circRNAs, predictedmiRNAs, and relevant biological processes were constructed to illustrate the molecular regulatory mechanism (Figures S5–S11).

## 4. Discussion

RNA-seq is an effective approach to elucidate the molecular functional properties of a wide variety of organisms by exploring their transcriptome profiling [11,22,23,24]. In this study, to further illustrate the biological events of skeletal muscle phenotypic differences between Beijing Black and Yorkshire pigs, the whole transcriptomes of the two breeds were compared and differentially expressed mRNAs, lncRNAs, and circRNAs were identified. Then, GO biological process and KEGG pathway analyses were performed to classify the differences in functions of those genes in the two pig breeds.

Myogenesis is a complex process including determination and proliferation of myoblasts, formation of myotubes and myofibers by fusion of myoblasts, and growth and maturation of myofibers [4,25]. Muscle mass is determined by the number and size of myofibers [26]. In pigs, the muscle growth is predominantly determined in prenatal periods and there are two waves of fiber generation with primary myofibers formation from 35 to 55 dpc (days post coitus) and followed by the formation of secondary myofibers which attach to primary myofibers between 50 and90 dpc [25,27,28]. Previous studies identified candidate genes regulating prenatal skeletal muscle development by RNA-seq or microarray methods [4,29,30]. Some reports have shown that there exists a third wave after birth and the size of muscle fibers reaches a plateau after 20 weeks [31,32]. The results of Zhao et al. demonstrate that differences in transcriptional profiling are more significant in postnatal periods than those in prenatal periods. Meanwhile, postnatal muscle growth is critical for the muscle phenotype difference between Lantang and Landrace pigs [33]. The transcriptome profiling of porcine skeletal muscle after birth has also been explored to identify the candidate genes relevant to skeletal muscle development [8,34,35]. In this study, SGCD, MSTN, MYOD1, PAX3, ITGA7, ITGB1BP2, IGF1, ANKRD2, SOX6, LMOD3, and CRYAB were enriched in the biological process of muscle organ development of adult Beijing Black and Yorkshire pigs. MSTN is a negative regulator for the growth and development of fetal and postnatal skeletal muscle mass, and inhibition of MSTN can increase muscle mass in animals [17,36,37,38]. Xu et al. showed that the expression of MSTN is decreased in the skeletal muscle of Wei pigs, an obese-type breed, at the adult stage compared to Yorkshire pigs, which suggests that this gene has less inhibition role in the muscle growth of Wei pigs than Yorkshire pigs at adult stage [8]. In agreement with observations of Xu et al., MSTN was more highly expressed in Yorkshire than in Beijing Black pigs in our analyses. IGF1 is necessary for cell proliferation and differentiation [39,40]. IGF1 is also critical for skeletal muscle development and can stimulate both proliferation and differentiation in myoblasts [18,41]. MYOD1, a transcriptional activator belonging to the basic helix-loop-helix family, can promote transcription of muscle-specific target genes and induce differentiation of fibroblasts into myoblasts [19]. MYOD1 is downregulated to a greater extent in obese-type Huainan pigs than in western commercial pigs. Compared to Mangalitsa pigs, Moravka pigs, which are characterized by a higher meat percentage and longer carcass with less fat, also show upregulated expression of MYOD1 [10]. In this study, IGF1 and MYOD1 showed higher expression in Yorkshire pigs, which might be relevant to their vigorous skeletal muscle growth. ANKRD2 belongs to the muscle ankyrin repeat protein family and is involved in the regulation of skeletal muscle cell differentiation [42]. Overexpression of ANKRD2 can inhibit C2C12 differentiation [43,44]. ANKRD2 is upregulated in obese-type Wei pigs compared to Yorkshire pigs [8]. This was coherent with the current study that Beijing Black pigs had a higher expression level of ANKRD2 than that in Yorkshire pigs, which contributed to high IMF content. CRYAB maintains the myoblasts in the proliferative stage and diminishes the formation of myotubes [45]. CRYAB was highly expressed in Beijing Black pigs compared to Yorkshire pigs, suggesting this gene inhibited the skeletal muscle growth in Beijing Black pigs. LMOD3, mainly expressed in skeletal muscle, belongs to the Leiomodin protein family [46,47]. Deletion of LMOD3 leads to inhibition of myogenic differentiation as MyoD, MyoG, and MyHC are significantly suppressed in C2C12 cells [48]. LMOD3 was associated with skeletal muscle development due to its more abundant level in Yorkshire pigs than Beijing Black pigs. Meanwhile, the differentially expressed genes, such as TRIM63, MYH8, MYH13, and MYLK2, were also involved in the muscle contraction. TRIM63, an E3 ubiquitin ligase that localized to the Z-line and M-line lattices of myofibrils, is related to muscle atrophy [49]. TRIM63 was highly expressed in Beijing Black pigs, which was coherent with the concept that TRIM63 was more expressed in the obese Basque pigs and contributed to the more tender meat as proteasome would promote post-mortem meat tenderization [50,51]. The extracellular matrix (ECM) and its receptors play essential roles in muscle development and maintenance [52]. Collagen is the essential component of the ECM and is related to pork tenderness [53]. COL1A2, COL3A1, COL5A1, and COL5A2 were more highly expressed in skeletal muscle of Yorkshire pigs than in those of Beijing Black pigs and are involved in biological processes focused on collagen fibril organization and extracellular matrix organization. Our results were consistent with those of previous studies in that the collagen genes were downregulated in obese pigs [8,11].

Beijing Black pigs have higher IMF content than Yorkshire pigs. The IMF content depends on the balance between synthesis and degradation of triglycerides [54]. The differentially expressed genes (FABP3, PDK4, CPT1A, LDLR, and UCP3) related to biological processes of long-chain fatty acid transport, regulation of fatty acid oxidation, cellular response to fatty acid, and fatty acid metabolic process contributed to the difference in IMF content between Beijing Black and Yorkshire pigs. FABP3 is a nuclear transcription factor that regulates the uptake of fatty acids and transport of them toward the mitochondrial β-oxidation system [55]. FABP3 promotes 3T3 cells differentiation and enhances triacylglycerol levels [56]. The polymorphisms of FABP3 are significantly associated with porcine IMF content [57,58,59]. The expression level of FABP3 in skeletal muscle was higher in obese pigs than that in lean pigs [8,11,54]. PDK4, a mitochondrial enzyme, is a member of the protein kinase family and is involved in the aerobic oxidation of carbohydrate fuels and fatty acids [60,61]. This gene is upregulated in obese pigs compared to lean pigs and the polymorphic site in PDK4 has a significant correlation with IMF content and muscle water content [8,11,62]. UCP3, a mitochondrial membrane protein, is involved in lipid metabolism. Overexpression of UCP3 can increase fatty acid oxidation in transgenic mouse skeletal muscle [21]. In a previous study, UCP3 gene polymorphisms were significantly associated with IMF content [63]. The UCP3 gene is more highly expressed in the skeletal muscle of obese pigs compared to lean pigs [8,64]. CPT1A is a rate-limiting enzyme and controls the entrance of fatty acids into the mitochondria for beta-oxidation [65]. Overexpression of the CPT1A enhances fatty acid oxidation in muscle cells [66]. CPT1A is upregulated in intramuscular adipose in obese pigs and performs a function in intramuscular adipose metabolism through the PPAR signaling pathway [67]. LDLR can regulate cholesterol homeostasis and lipid metabolism. The expression of LDLR reduces lipolysis and promotes lipid storage in skeletal muscles of obese pigs due to its downregulated expression level [8]. In our study, FABP3, PDK4, UCP3, and CPT1A were highly expressed, while LDLR was expressed at a low level in Beijing Black pigs, which is in conformance with previous reports and can be deduced that these differentially expressed genes contributed to intramuscular lipid deposition in Beijing Black pigs.

LncRNAs can regulate the expression of protein-coding genes via both *cis* and *trans*-acting mechanisms [68,69]. Accumulating evidence suggests that lncRNAs play vital roles in many biological processes such as immune response and metabolism [70,71]. In this study, a total of 322 differentially expressed lncRNAs were obtained, and 483 *cis*-target genes together with 4173 *trans*-target genes were predicted. The results of functional analysis implied that differentially expressed lncRNAs were involved in the regulation of skeletal muscle development, fat cell differentiation, and fatty acid metabolism. CSRP3 is a cofactor for myogenic bHLH transcription factors and promotes myoblast differentiation [72,73]. It is upregulated during porcine embryonic primary and secondary fiber formation and promotes more rapid body growth in Yorkshire pigs [11,74]. CSRP3 was predicted to be *trans*-targeted by XLOC_243473, XLOC_246104, XLOC_240533, XLOC_100917, XLOC_240947, XLOC_021126, and XLOC_127634. MYF6, also called MRF4, is a bHLH DNA binding protein involved in myofiber formation during the early stages of myogenesis and is implicated as a muscle determination factor in the absence of MYOD1 and MYF5 [75,76]. The differentially expressed lncRNA, XLOC_178274, XLOC_161639, XLOC_233142, XLOC_196082, XLOC_084900, XLOC_163479, XLOC_094551, XLOC_054069, XLOC_024423, XLOC_060910, XLOC_251721, XLOC_061619, and XLOC_113404, acted on MYF6. IGFBP-5 is expressed in the developing myotome [77]. It has the potential to regulate the proliferation of embryonic myogenic cells and influences postnatal muscle mass [78]. This gene was regulated by XLOC_244862, XLOC_246105, XLOC_188651, XLOC_247320, XLOC_240975, XLOC_047148, XLOC_246055, XLOC_184037, and XLOC_240947. DCN, a small proteoglycan in the extracellular matrix, can interact with TGFB1 to induce myogenic satellite cell proliferation and differentiation [79]. The differentially expressed XLOC_242810, XLOC_244900, XLOC_240947, XLOC_193144, XLOC_218131, and XLOC_242815 regulated myogenesis by DCN. Moreover, some target genes were clustered in the pathways related to skeletal muscle development in this study. MAP2K1 and MRAS were involved in the MAPK signaling pathway, while MAP2K1 was also involved in the PI3K-Akt signaling pathway together with SPP1. MAP2K1 can activate MEF2A, MEF2C, and MYOD1 and promote myoblast differentiation [80,81]. This gene was targeted by XLOC_047147 and XLOC_242812. MRAS, a member of the Ras family small GTPases, was regulated by XLOC_000018 and XLOC_112338. It acts as a negative regulator of myoblast differentiation [82]. During the process of skeletal muscle regeneration, SPP1 is upregulated and is an important mediator in the early phase of muscle regeneration as it promotes macrophage binding to necrotic fibers [83]. XLOC_020703, XLOC_153228, XLOC_141271, XLOC_166158, XLOC_105481, and XLOC_246046 playeda role in skeletal muscle development by altering the expression of SPP1. PPARD belongs to the nuclear hormone receptor superfamily and plays an essential role in adipogenesis and lipid metabolism [84,85,86]. XLOC_082134 and XLOC_218131 were deduced to regulate fatty acid beta-oxidation and adipose tissue development between Beijing Black and Yorkshire pigs through PPARD. FOXO1, a member of the transcription factor FOXO family, can regulate adipocyte differentiation [87]. It was the *trans*-target gene of XLOC_146723, XLOC_244908, XLOC_176687, XLOC_241005, XLOC_246783, and XLOC_245711. ACSL1 plays a key role in lipid biosynthesis and fatty acid degradation. It facilitates the uptake of fatty acid in 3T3-L1 adipocytes [88]. XLOC_242810, XLOC_093511, XLOC_156330, and XLOC_203876 were predicted to target ACSL1 and are responsible for fatty acid metabolism. LPIN1 is required for adipocyte differentiation and lipid metabolism, whereas adipocyte differentiation is impaired in lipin-deficient mice [89]. This gene was related to the biological process of triglyceride biosynthesis under the regulation of XLOC_240975, XLOC_153228, and XLOC_240947 in this study.

CircRNA, characterized by a closed-loop structure through a 5′ to 3′ phosphodiester bond, is a type of single-stranded RNA generated by pre-mRNA back splicing [90]. CircRNA plays a key role in the regulation of myoblast differentiation and adipogenic differentiation of adipose-derived stem cells [91,92]. To shed light on the potential function of circRNAs in porcine skeletal muscle-related traits in obese and lean pigs, the host genes of differentially expressed circRNAs were selected to perform GO enrichment analysis. The differentially expressed novel_circ_0006903 (ACTA1), novel_circ_0006919 (ACTA1), novel_circ_0006915 (ACTA1), novel_circ_0023543 (MYBPC2), novel_circ_0007768 (MYL1), novel_circ_0008800 (NEB), and novel_circ_0018632 (DYSF) were related to muscle contraction and muscle filament sliding given their host genes. As circRNA can act as miRNA sponges [16], the binding potentials of miRNAs to circRNAs were predicted. The differentially expressed genes related to skeletal muscle development and fatty acid metabolism were responsible for skeletal muscle-related traits, therefore, the crosstalks among differentially expressed circRNAs, predicted miRNA, and candidate regulatory genes were constructed. miR-206 is specifically expressed in skeletal muscle and associated with the development stage [93]. It can also target IGF1 and regulate tilapia growth [94]. In this study, novel_circ_0017816 and novel_circ_0026337 were predicted to interact with miR-206 and regulated the expression of IGF1. miR-199a-3p can target IGF1 and modulate the IGF-1/AKT/mTOR pathway to inhibit C2C12 myogenic differentiation [95]. Novel_circ_0011583 influenced the expression of IGF1 as it abolished the inhibitory effect of miR-199a-3p by its ceRNA role. miR-221 can target MYOD1 and suppresses its expression in C2C12 cells [96]. This miRNA was predicted to bind with novel_circ_0011583 and took part in the regulation of MYOD1 expression.

A set of differentially expressed mRNAs, lncRNAs, and circRNAs involved in the regulation of skeletal muscle phenotypes were identified in Beijing Black and Yorkshire pigs. However, several limitations in this study should be noted. First, only the information of the transcriptome was profiled in this study. The expression and modification of proteins that directly influence performance traits were not studied. Second, the potential IMF-related genes were only identified from the samples of skeletal muscle as the IMF tissues were hard to collect and purify. Finally, further studies should be performed to illustrate the molecular mechanisms of the candidate genes.

## 5. Conclusions

This study characterized the expression profiles of mRNAs, lncRNAs, and circRNAs in skeletal muscles of Beijing Black and Yorkshire pigs. Functional enrichment analysis suggested that differentially expressed mRNAs influenced the growth rate and IMF content by regulating skeletal muscle development and fatty acid metabolism. Differentially expressed lncRNAs and circRNAs also took part in the regulation of skeletal muscle development and fatty acid metabolism by their *cis*/*trans*-target genes or circRNA-miRNA-mRNA interactions. The current work provides a set of valuable candidate factors related to muscle phenotypic variation in different pig breeds and adds new insights into the understanding of the molecular regulatory mechanism for porcine skeletal muscle growth and meat quality.

## Data Availability

All the data used in this study were submitted to the Sequence Read Archive database with accession number PRJNA736938 and will be released on 18 July 2022 (https://www.ncbi.nlm.nih.gov/sra).

## Supplementary Materials

The following are available online at https://www.mdpi.com/article/10.3390/ani11113169/s1, Figure S1: Genomics distribution of clean reads in eight libraries; Figure S2:Correlation of the samples; Figure S3: Identification and characterization of lncRNAs in porcine skeletal muscle; Figure S4: Identification and characterization of circRNAs in porcine skeletal muscle; Figure S5:Interaction network related to muscle organ development; Figure S6: Interaction network related to extracellular matrix organization; Figure S7: Interaction network related to collagen fibril organization; Figure S8: Interaction network related to long-chain fatty acid transport; Figure S9: Interaction network related to regulation of fatty acid oxidation; Figure S10: Interaction network related to cellular response to fatty acid; Figure S11: Interaction network related to fatty acid metabolic process; Table S1: Summary of sequencing data; Table S2: Mapping information of clean reads against the porcine reference genome; Table S3: Summarization of identified lncRNAs in porcine skeletal muscles; Table S4: Summarization of identified circRNAs in porcine skeletal muscles; Table S5: Differentially expressed mRNAs, lncRNAs, and circRNAs in skeletal muscle between Beijing Black and Yorkshire pigs; Table S6: GO and KEGG pathway enrichment analysis of differentially expressed mRNAs; Table S7: The potential *cis* and *trans*-targets of lncRNAs; Table S8: GO and KEGG pathway enrichment analysis of target genes regulated by differentially expressed lncRNAs; Table S9: GO and KEGG pathway enrichment analysis of the host genes generating differentially expressed circRNAs; Table S10: Predicted miRNAs interacted with differentially expressed circRNAs.
