# Supplementary material for: Genome-Wide Expression Profiling of mRNAs, lncRNAs and circRNAs in Skeletal Muscle of Two Different Pig Breeds
Source: Animals (Basel). 2021 Nov 5;11(11):3169. doi: 10.3390/ani11113169 (PMC8614396; doi:10.3390/ani11113169)
Supplement: Supplementary file 1 [file animals-11-03169-s001.zip › Illustration of supplementary figures.pdf]

Figure S1. Genomics distribution of clean reads in eight libraries.

Figure S2. Correlation of the samples. B represented Beijing Black pigs. Y represented Yorkshire pigs.

Figure S3. Identification and characterization of lncRNAs in porcine skeletal muscle. (A) Venn diagram of lncRNAs with coding potential. (B) Comparison of transcript length between mRNAs and lncRNAs. (C) Comparison of exon number between mRNAs and lncRNAs. (D) Comparison of ORF length between mRNAs and lncRNAs.

Figure S4. Identification and characterization of circRNAs in porcine skeletal muscle. (A) Genomic origin of circRNAs. (B) Length distribution of circRNAs. (C) Chromosome distribution of circRNAs.

Figure S5. Interaction network related to muscle organ development. Orange rounded rectangles indicate GO terms. Green circles indicate mRNAs. Blue rhombuses indicate lncRNAs. Pink circles indicate circRNAs. Red triangles indicate miRNAs.

Figure S6. Interaction network related to extracellular matrix organization. Orange rounded rectangles indicate GO terms. Green circles indicate mRNAs. Blue rhombuses indicate lncRNAs. Pink circles indicate circRNAs. Red triangles indicate miRNAs.

Figure S7. Interaction network related to collagen fibril organization. Orange rounded rectangles indicate GO terms. Green circles indicate mRNAs. Blue rhombuses indicate lncRNAs. Pink circles indicate circRNAs. Red triangles indicate miRNAs.

Figure S8. Interaction network related to long-chain fatty acid transport. Orange rounded rectangles indicate GO terms. Green circles indicate mRNAs. Blue rhombuses indicate lncRNAs. Pink circles indicate circRNAs. Red triangles indicate miRNAs.

Figure S9. Interaction network related to regulation of fatty acid oxidation. Orange rounded rectangles indicate GO terms. Green circles indicate mRNAs. Blue rhombuses indicate lncRNAs. Pink circles indicate circRNAs. Red triangles indicate miRNAs.

Figure S10. Interaction network related to cellular response to fatty acid. Orange rounded rectangles indicate GO terms. Green circles indicate mRNAs. Blue rhombuses indicate lncRNAs. Pink circles indicate circRNAs. Red triangle indicate miRNAs.

Figure S11. Interaction network related to fatty acid metabolic process. Orange rounded rectangles indicate GO terms. Green circles indicate mRNAs. Blue rhombuses indicate lncRNAs. Pink circles indicate circRNAs. Red triangles indicate miRNAs.
