# Supplementary figures and images for: Genome-Wide Expression Profiling of mRNAs, lncRNAs and circRNAs in Skeletal Muscle of Two Different Pig Breeds
Source: Animals (Basel). 2021 Nov 5;11(11):3169. doi: 10.3390/ani11113169 (PMC8614396; doi:10.3390/ani11113169)

**Y1**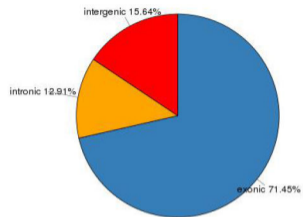**Y2**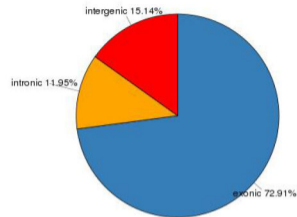**Y3**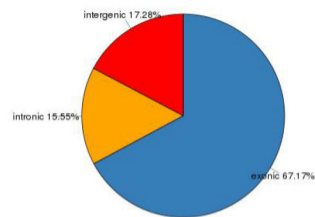**Y4**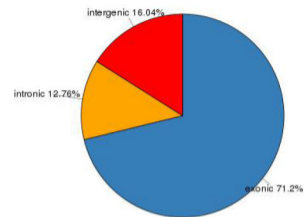**B1**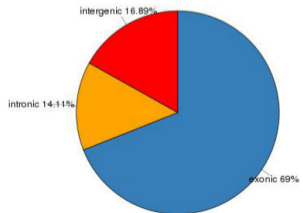**B2**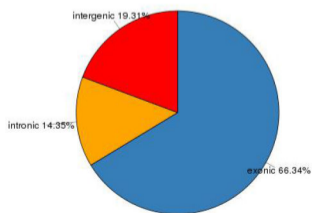**B3**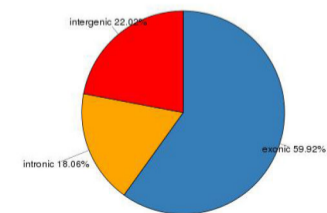**B4**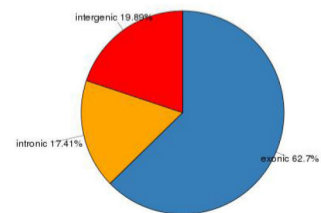

Supplement: Supplementary file 1 [file animals-11-03169-s001.zip › FigureS1.pdf]

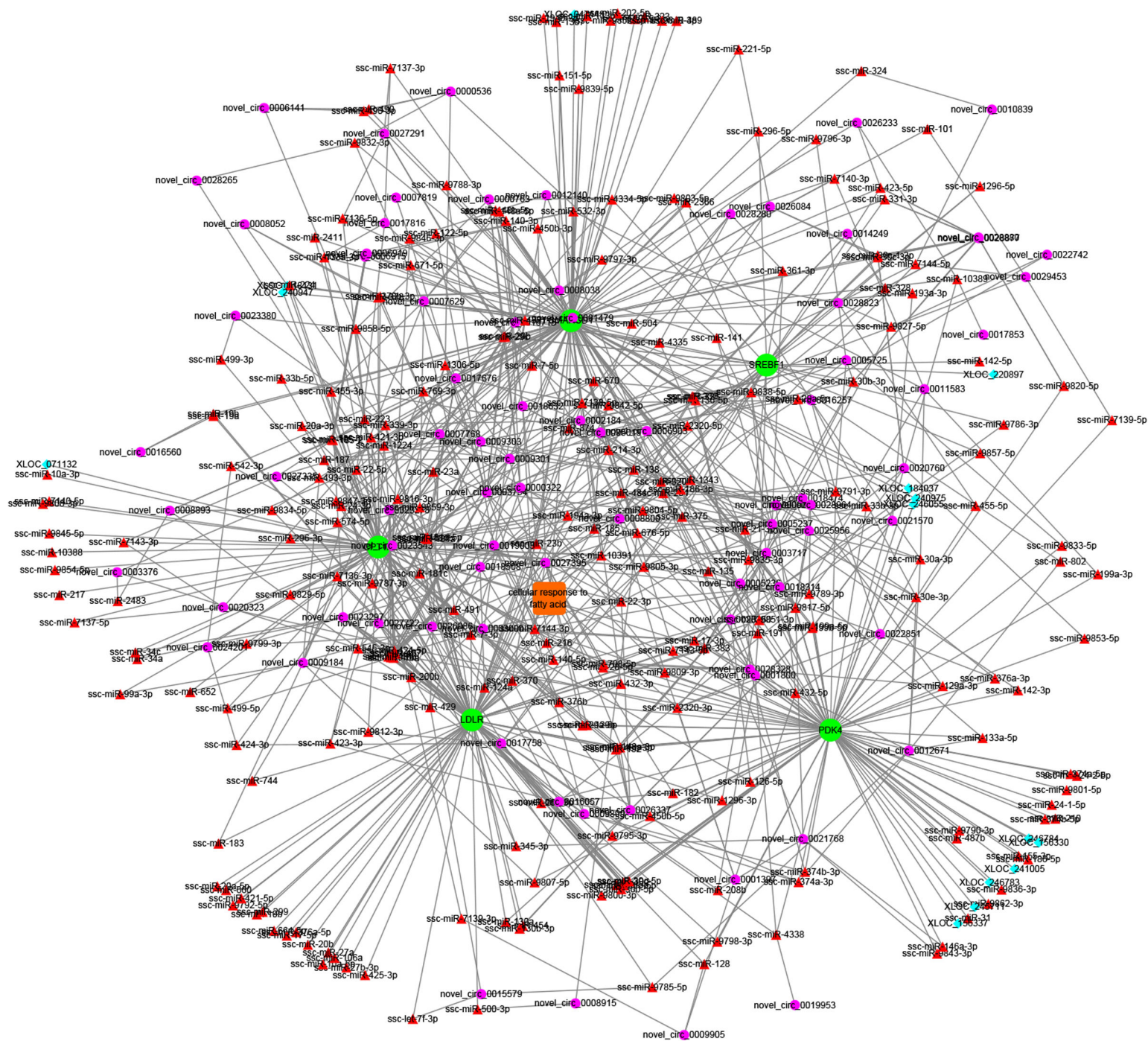

Supplement: Supplementary file 1 [file animals-11-03169-s001.zip › FigureS10.pdf]

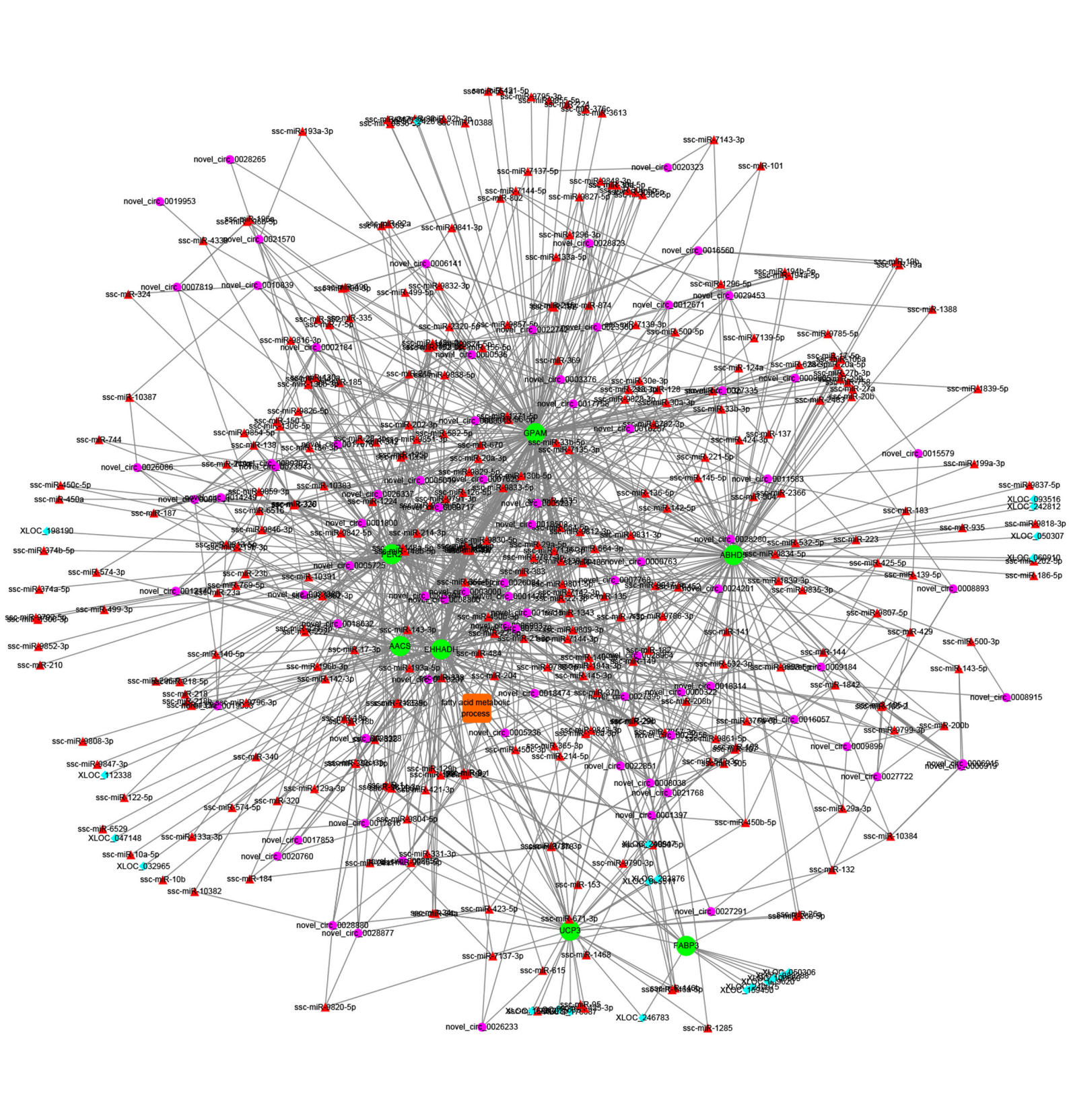

Supplement: Supplementary file 1 [file animals-11-03169-s001.zip › FigureS11.pdf]

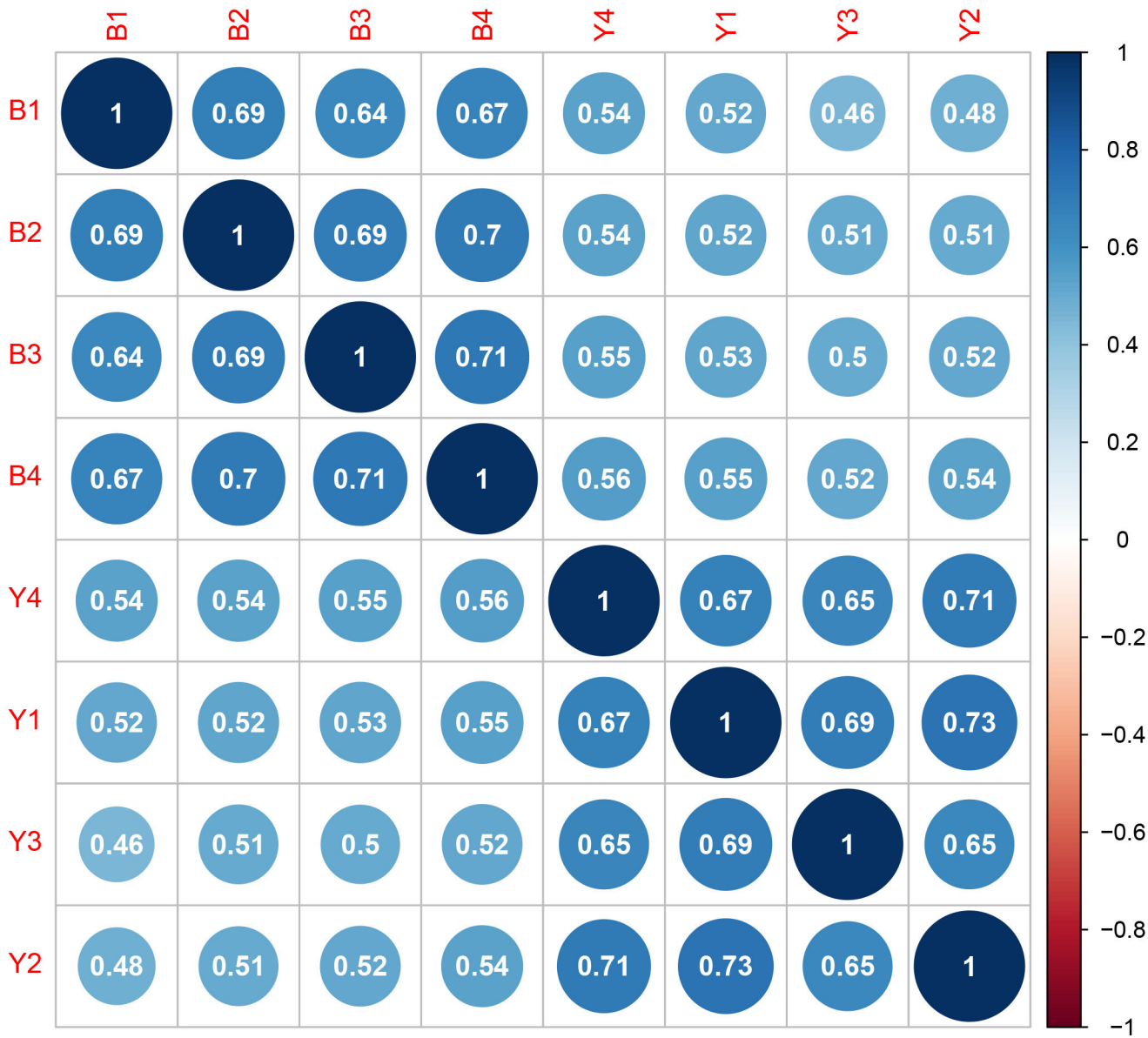

Supplement: Supplementary file 1 [file animals-11-03169-s001.zip › FigureS2.pdf]

**A**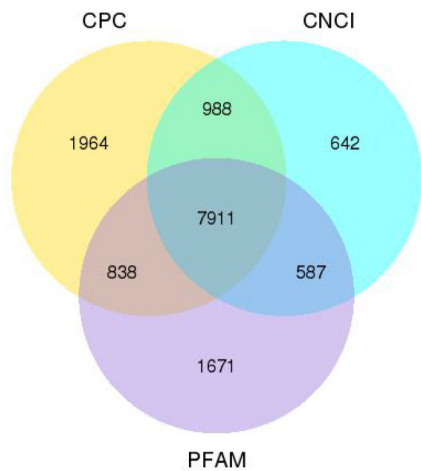**B**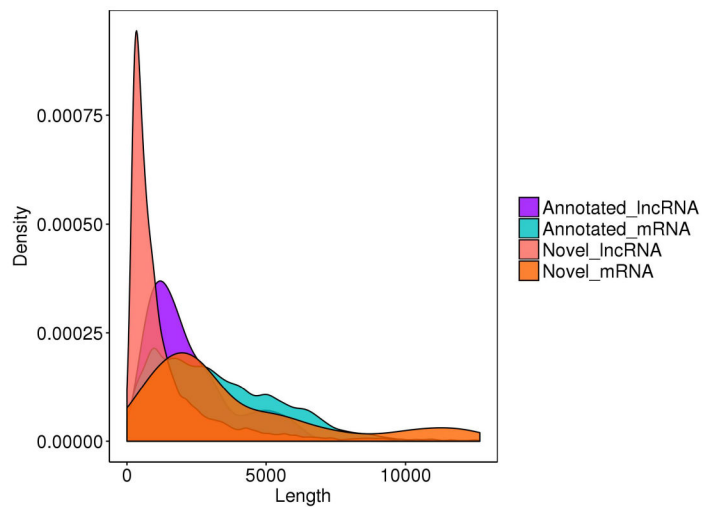**C**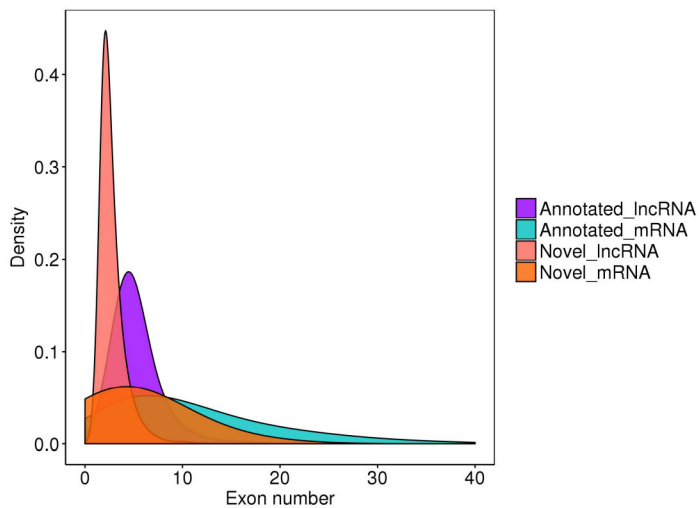**D**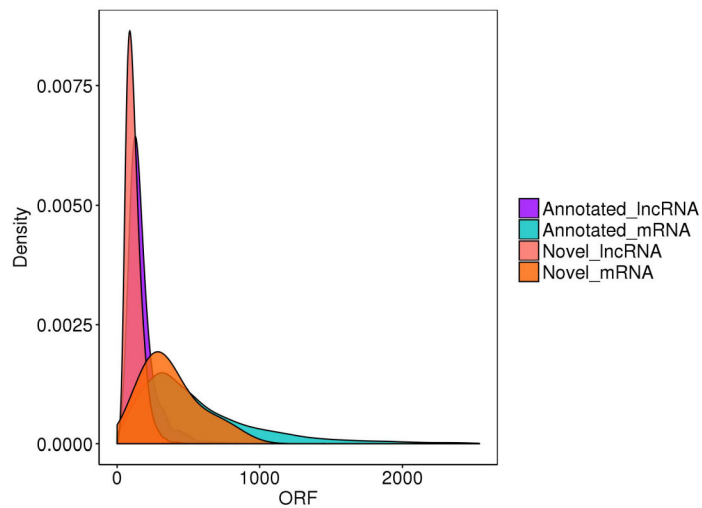

Supplement: Supplementary file 1 [file animals-11-03169-s001.zip › FigureS3.pdf]

**A**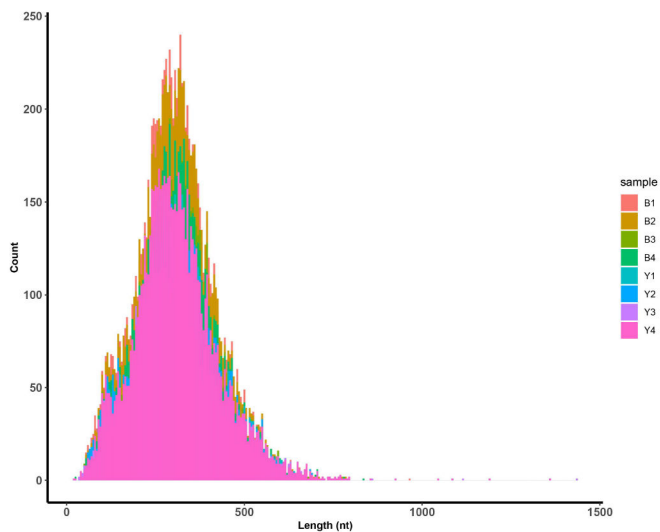**B**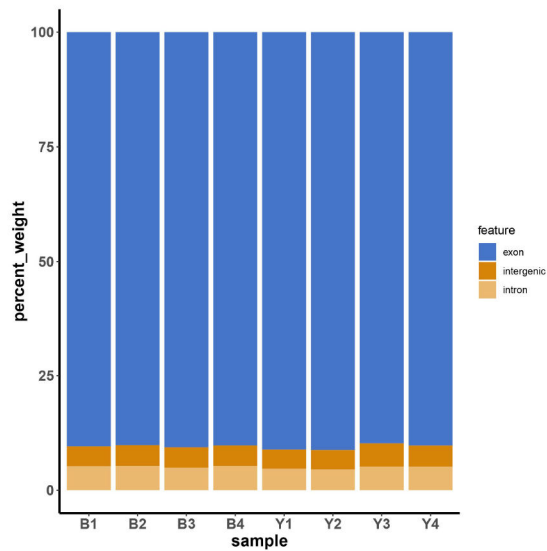**C**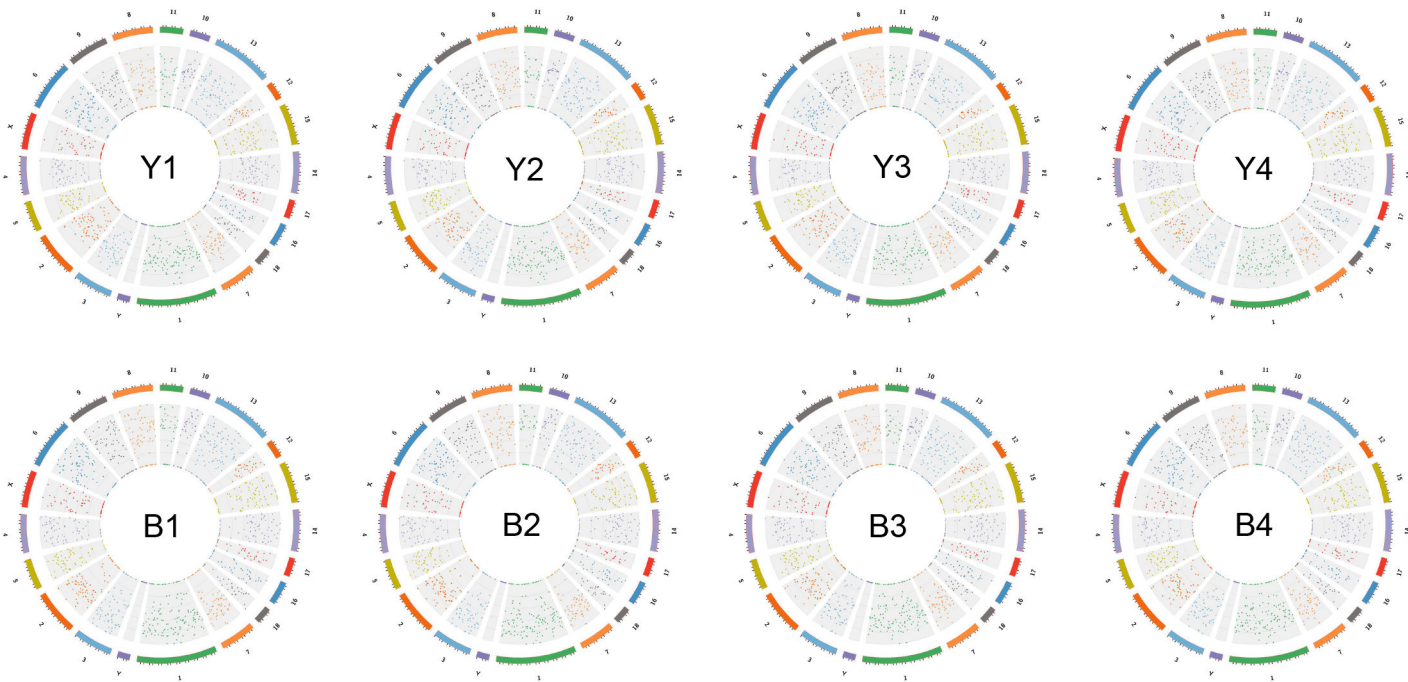

Supplement: Supplementary file 1 [file animals-11-03169-s001.zip › FigureS4.pdf]

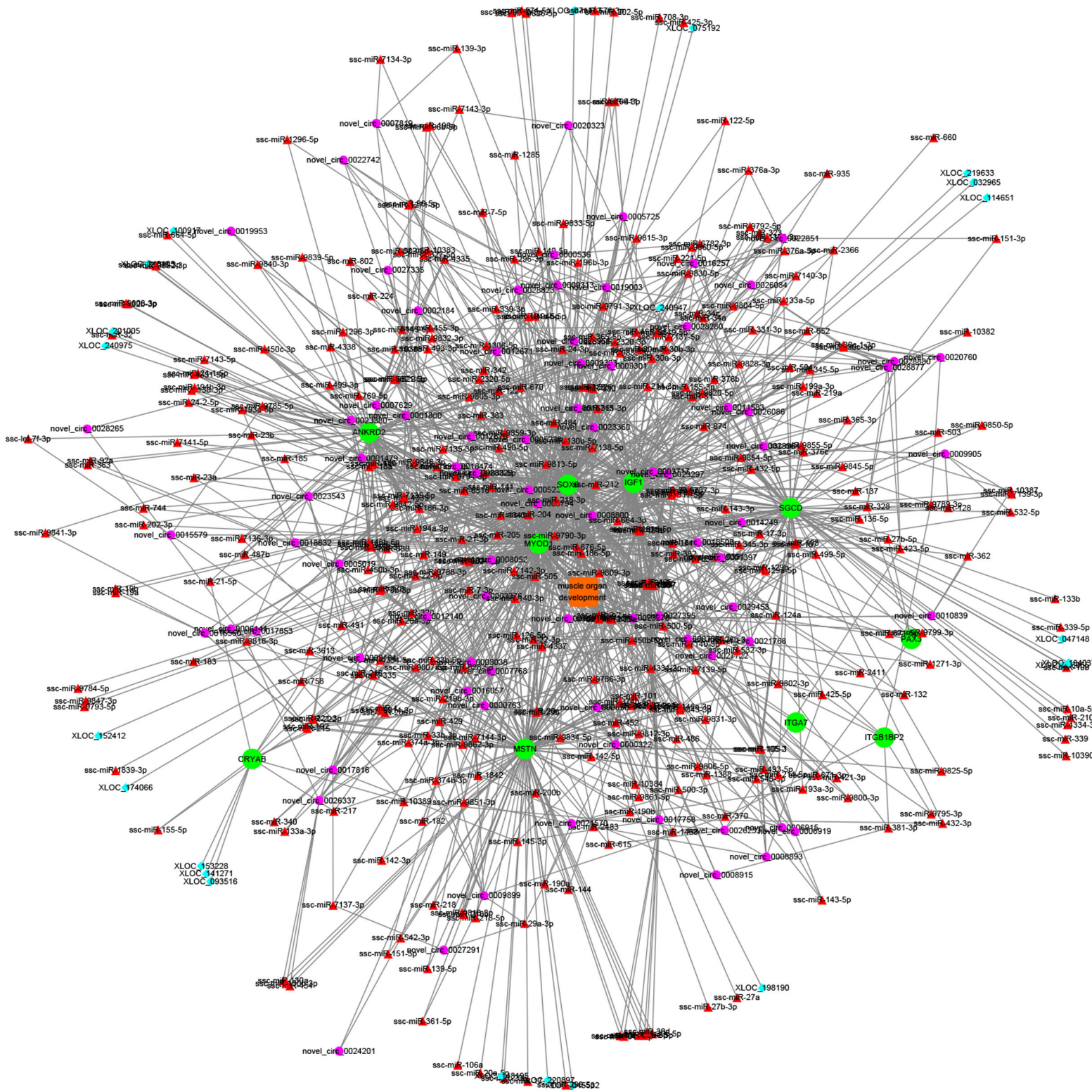

Supplement: Supplementary file 1 [file animals-11-03169-s001.zip › FigureS5.pdf]

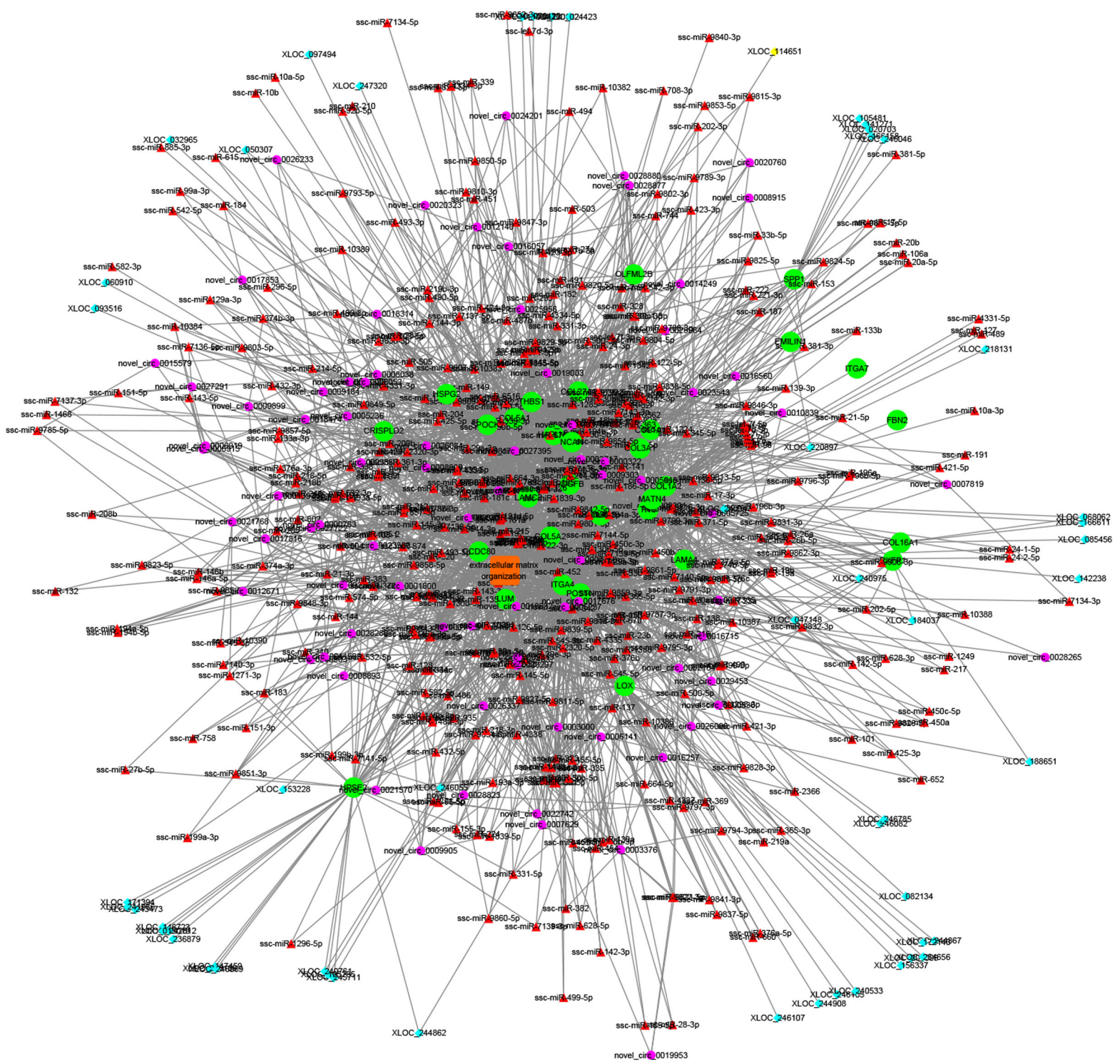

Supplement: Supplementary file 1 [file animals-11-03169-s001.zip › FigureS6.pdf]

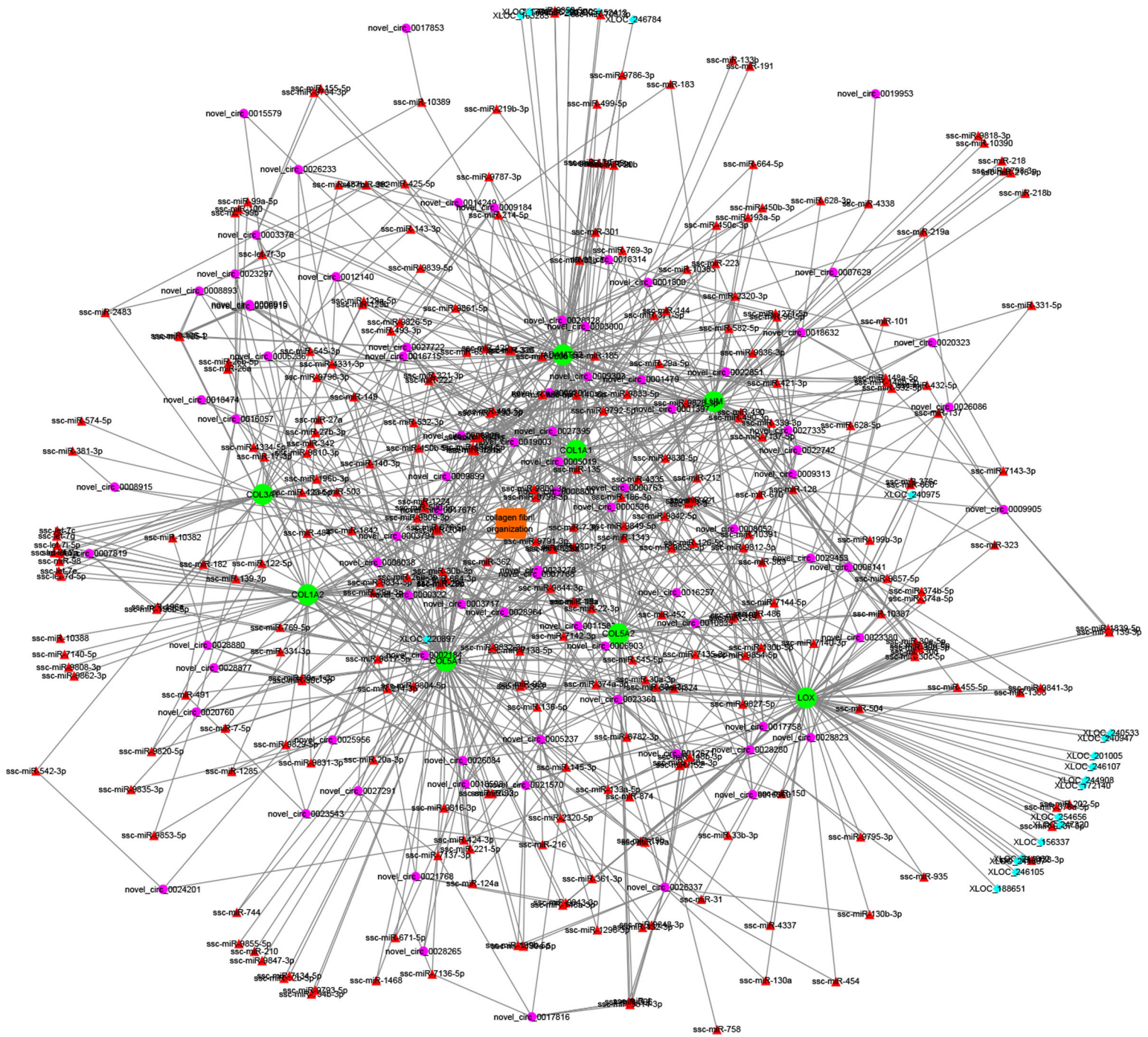

Supplement: Supplementary file 1 [file animals-11-03169-s001.zip › FigureS7.pdf]

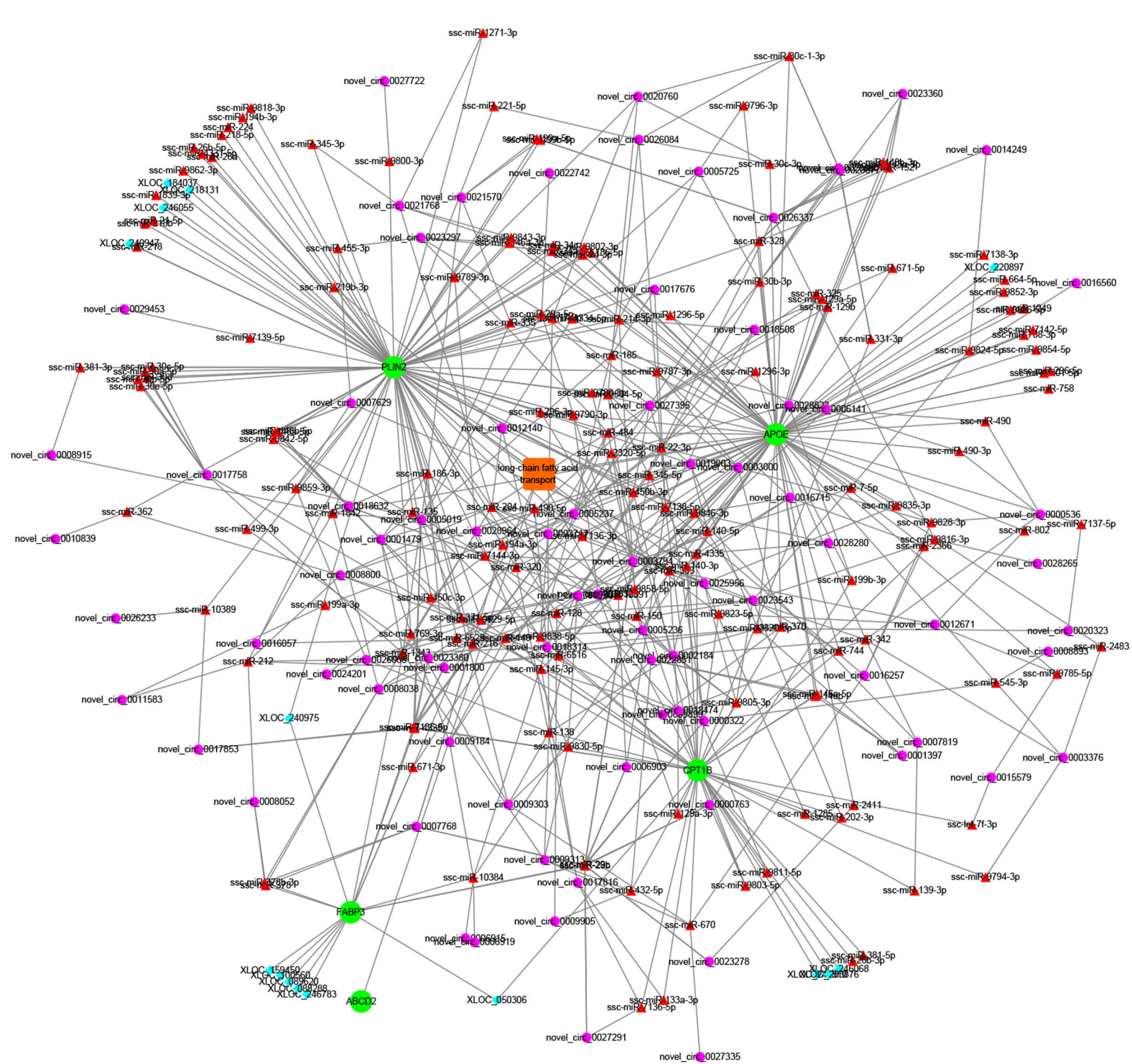

Supplement: Supplementary file 1 [file animals-11-03169-s001.zip › FigureS8.pdf]

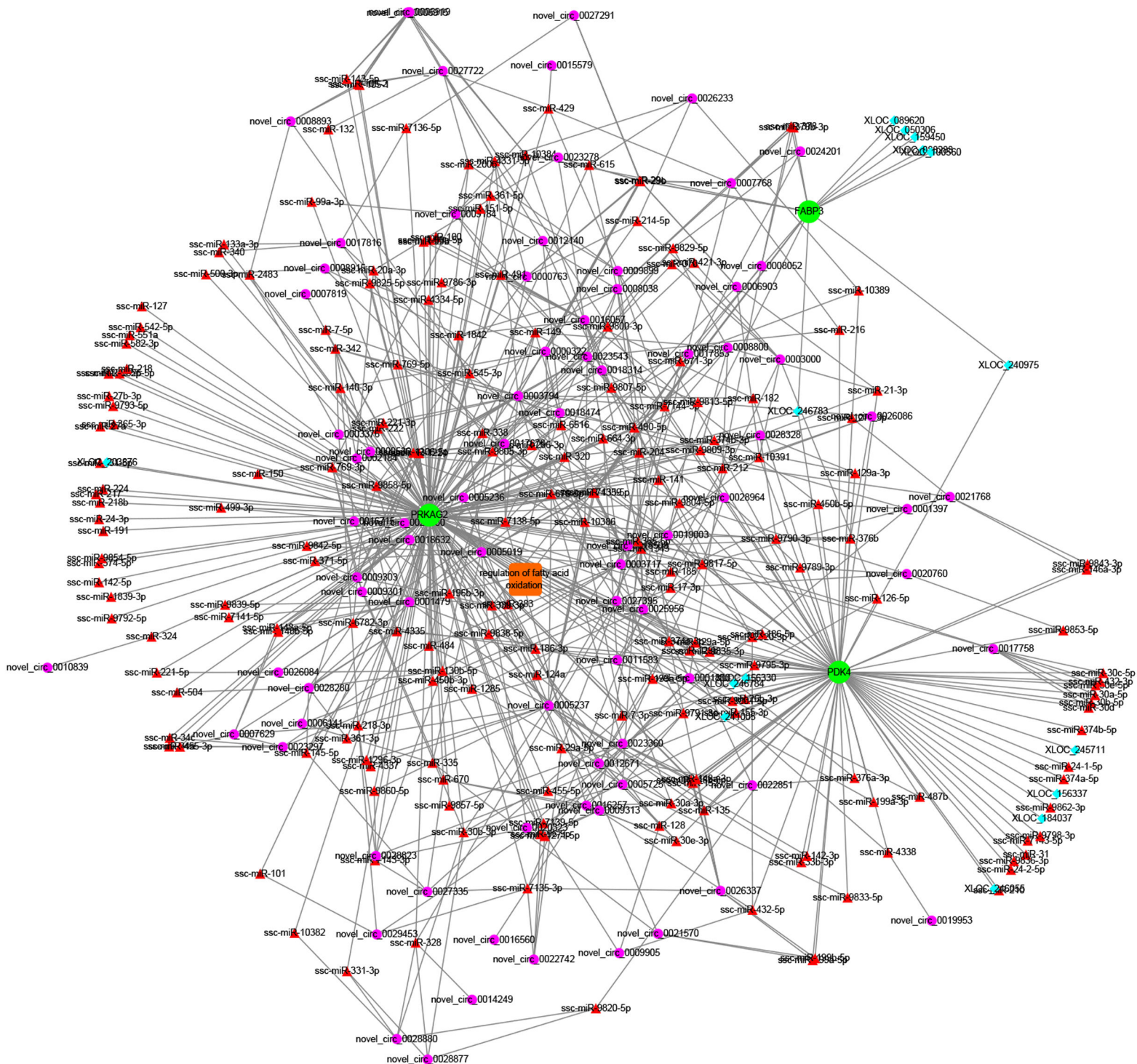

Supplement: Supplementary file 1 [file animals-11-03169-s001.zip › FigureS9.pdf]
